# Supplementary figures and images for: Stochastic dispersal increases the rate of upstream spread: A case study with green crabs on the northwest Atlantic coast
Source: PLoS One. 2017 Sep 29;12(9):e0185671. doi: 10.1371/journal.pone.0185671 (PMC5621684; doi:10.1371/journal.pone.0185671)

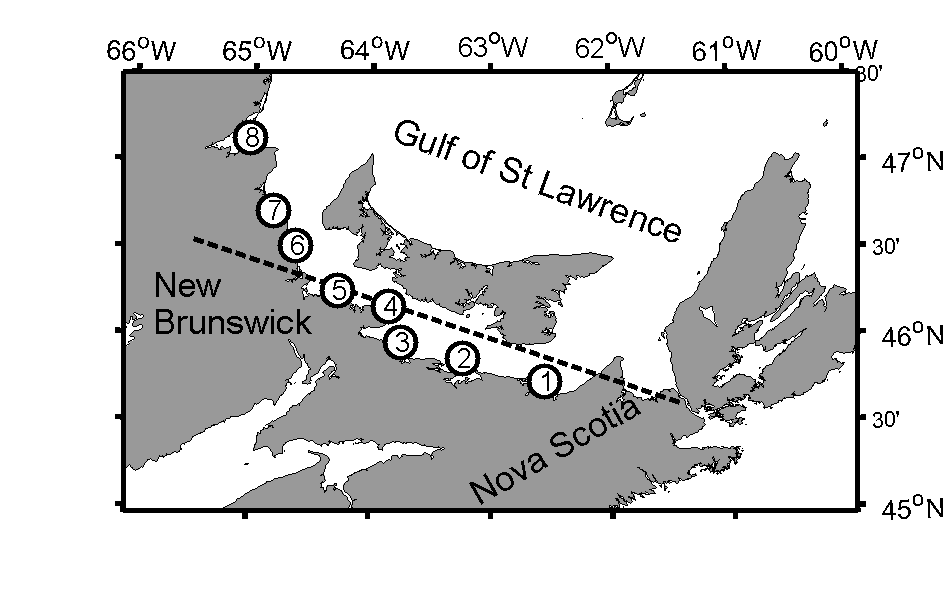

Supplement: S1 Fig — The locations are numbered from 1 to 8, from the southeast to the northwest. The dashed line is the modeled coastline used for calculating dispersal displacement of projected particles. (TIF) [file pone.0185671.s001.tif]

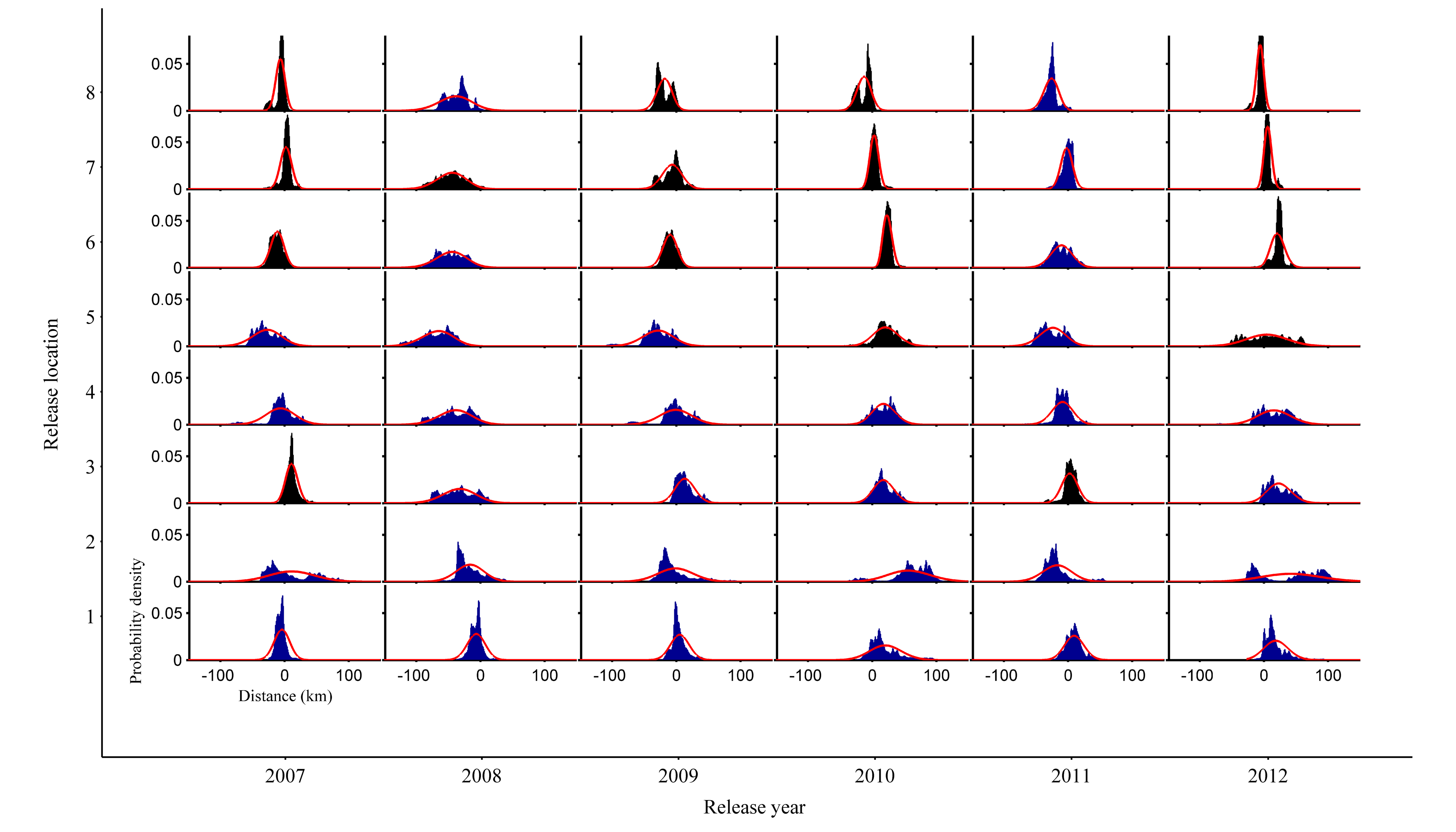

Supplement: S2 Fig — Larval dispersal kernels obtained from simulations of the hydrodynamic model of the Gulf of St. Lawrence [20,21] for different locations in the Northumberland Strait (see S1 Fig) and different years. For a given panel, the frequency distribution of the displacements of dispersing particles (representing green crab larvae) is in blue, and the fitted theoretical dispersal kernel (Eqs 3–5) is in red. See S1 Table for the estimated values of the pairs of v (net rate of displacement) and D (diffusion coefficient) corresponding to each simulated kernel. (TIF) [file pone.0185671.s002.tif]
