# Supplementary material for: Stochastic dispersal increases the rate of upstream spread: A case study with green crabs on the northwest Atlantic coast
Source: PLoS One. 2017 Sep 29;12(9):e0185671. doi: 10.1371/journal.pone.0185671 (PMC5621684; doi:10.1371/journal.pone.0185671)
Supplement: S1 Table — Pairs of (v,D) for larval dispersal kernels simulated from the hydrodynamic model for the Gulf of St. Lawrence and then estimated using the fitted Eqs 3–5 (see also S1 and S2 Figs). Units of the net rate of displacement v and diffusion coefficient D are km d-1 and km2 d-1, respectively. (PDF) [file pone.0185671.s003.pdf]

Location	Year 2007	2008	2009	2010	2011	2012
8	(-0.0953, 0.368)	(-0.565, 4.53)	(-0.271,0.923)	(-0.188,0.844)	(-0.383,0.824)	(-0.090,0.225)
7	(0.0317, 0.574)	(-0.652, 3.35)	(-0.0959, 1.7)	(0.0448,0.34)	(-0.033,0.602)	(0.083, 0.252)
6	(-0.165, 0.753)	(-0.654, 3.64)	(-0.144,0.913)	(0.339, 0.239)	(-0.151, 2.02)	(0.304, 0.779)
5	(-0.398, 3.62)	(-0.965, 3.46)	(-0.419, 3.97)	(0.303, 2.76)	(-0.351, 2.89)	(0.0624, 7.55)
4	(-0.0926, 3.81)	(-0.569, 4.51)	(-0.012, 4.64)	(0.26, 2.29)	(-0.129, 1.96)	(0.227, 4.92)
3	(0.159, 0.63)	(-0.475, 4.77)	(0.195, 1.66)	(0.258, 1.84)	(0.0357, 1.15)	(0.337, 2.51)
2	(0.163, 9.42)	(-0.239, 3.44)	(-0.018, 5.72)	(0.833, 7.48)	(-0.249, 3.7)	(0.614, 15.8)
1	(-0.0602, 1.09)	(-0.096, 1.48)	(0.0784, 1.57)	(0.302, 4.58)	(0.138, 1.7)	(0.27, 2.63)
